# Psycho-Emotional Approach to the Psychological Distress Related to the COVID-19 Pandemic in Spain: A Cross-Sectional Observational Study

**DOI:** 10.3390/healthcare8030190

**Published:** 2020-06-28

**Authors:** Sara Domínguez-Salas, Juan Gómez-Salgado, Montserrat Andrés-Villas, Diego Díaz-Milanés, Macarena Romero-Martín, Carlos Ruiz-Frutos

**Affiliations:** 1 Department of Psychology, Universidad Loyola Andalucía, Dos Hermanas, 41704 Sevilla, Spain; sdominguez@uloyola.es; 2 Department of Sociology, Social Work and Public Health, Faculty of Labour Sciences, University of Huelva, 21007 Huelva, Spain; salgado@uhu.es (J.G.-S.); frutos@uhu.es (C.R.-F.); 3 Safety and Health Postgraduate Programme, Universidad Espíritu Santo, Guayaquil 092301, Ecuador; 4 Department of Social, Developmental and Educational Psychology, Faculty of Education, Psychology, and Sports Sciences, University of Huelva, 21007 Huelva, Spain; 5 Department of Nursing, Faculty of Nursing, University of Huelva, 21071 Huelva, Spain; macarena.romero@denf.uhu.es

**Keywords:** COVID-19, psychological distress, pandemic, disease prevention, mental health, public health, novel coronavirus

## Abstract

Anxiety, depression, and stress are common and expected reactions to the coronavirus disease (COVID-19) pandemic. The objective of this study is to analyze psychological distress in a sample of Spanish population, identifying the predictive nature of the information received, the preventive measures taken, level of concern, beliefs, and knowledge about the infection. A cross-sectional observational study was conducted on a sample of 4615 participants. Data were collected through a self-prepared questionnaire and the general health questionnaire (GHQ-12). Bivariate analyses and logistic regressions were performed. Of the total participants, 71.98% presented psychological distress. The study population actively sought information about coronavirus, expressed a high level of concern and knowledge, and the most frequent preventive behavior was hand washing. As predictive factors, the degree of concern for COVID-19 was identified (odds ratio (OR) = 1.244, 95% confidence interval (CI) = [1.179, 1.312]), the number of hours spent consulting information on COVID-19 (OR = 1.038, 95% CI = [1.009, 1.068]), or the need for psychological support (OR = 1.135, 95% CI = [1.094, 1.177]), among others. These results could help design more effective strategies towards a psycho-emotional approach for the population when in similar health crisis situations. There is a need for interventions aimed at the psychological well-being of the population that meet the needs of their reality.

## 1. Introduction

The World Health Organization (WHO), on 11 March, classified the health crisis triggered by coronavirus disease (COVID-19) as the 2020 pandemic in the face of 118,000 reported cases and 4291 deaths in 114 countries [1]. In Spain, the state of health alert was declared on 14 March 2020 [2], involving a reduction of free movement of citizens. As a measure for virus containment and protection of the population, people were forced to remain confined at home, face-to-face educational activities were cancelled, as well as cultural, leisure and sports or religious activities, including funerals and all commercial activity except for that related to essential goods. The rapid spread of the disease forced, two weeks later, to increase restrictions by limiting activity to those considered essential, which are: health workers; pharmaceuticals; optician and orthopedic products; R&D&I (Research and Development and Innovation); and biotech centers linked to COVID-19; police forces; armed forces; civil protection; firefighting; traffic and road safety; private security; security transport; persons serving the elderly, dependent, or disabled; hygiene products; press and stationery; gas stations; tobacconists; technological and telecommunications equipment; food and beverages; supply of basic necessities; pet food; dry cleaners and laundromats; electronic commerce; telephone or correspondence [3].

Severe acute respiratory syndrome coronavirus 2 (SARS-COV-2) virus belongs to the Coronaviridae family and produces a clinical picture called coronavirus disease 2019 (COVID-19) [4]. It is believed that the main routes of transmission between people are through secretions (nasopharyngeal and saliva) [5], by contact with drops from the nose and mouth that occur when coughing or exhaling, or through contact with contaminated inert objects and hands that reach the mucous membrane of the mouth, nose, and eyes [4,6,7]. The groups considered most at risk are people over the age of 60, people with cardiovascular diseases and high blood pressure, diabetes, chronic lung disease, cancer, immunosuppression, or who are pregnant [4]. The most common symptoms and signs identified are: fever, dry cough, asthenia, dyspnoea, expectoration, sore throat, headache, myalgia or arthralgia, chills, nausea or vomiting, nasal congestion, diarrhea, hemoptysis, and conjunctival congestion [8,9].

Evidence suggests that anxiety, depression and stress are common and expected reactions to the COVID-19 pandemic [10]. Recent studies on the effect of similar pandemics on the population indicate that the factors that have contributed the most to reducing the psychological impact of isolation at home were to have received clear and consistent information [11,12,13]; explanation on the reasons for isolation and its necessity [14,15]; or having social, moral and economic support, as well as the absence of new contagions [16]. On the other hand, providing information to the population reduces their perception of risk to an epidemic [17].

A study conducted at the onset of the pandemic revealed public satisfaction with the available information on COVID-19, although developments updates were associated with lower levels of anxiety, especially with regard to the routes of transmission, the availability of a treaty or vaccine, and the number of affected people [18]. However, another study found that the time spent on information on COVID-19 contributed to psychological distress and increased feelings of loneliness [19], as well as the amount of time thinking about coronavirus [20].

Since no vaccine or specific treatment is available for COVID-19, the only way of protection for the population is to avoid exposure to the virus [21]. As personal prevention measures, it is recommended to use masks, respiratory etiquette, frequent hand hygiene, avoiding touching the eyes, avoiding public contact, maintaining a safe distance between people, and cleaning and disinfecting of the nearby environment [8]. Collective prevention measures such as confinement and social isolation have been shown to be effective in reducing the spread of the virus [22], decreasing the number of cases [23,24,25]. 

The knowledge, beliefs, and concern of the population play an important role in controlling the spread of disease. Concerns about infection, perception of the effectiveness of measures, and assessment of the usefulness of the information provided [26] have been identified as influencing factors for adherence to personal preventive measures. The greater the people’s knowledge about the disease, the greater perception of risk and adherence to prevention measures [27]. People who are well aware of the routes of transmission of contagious diseases are more likely to take preventive measures [27,28,29]. On the other hand, having more knowledge about the disease decreases the concern about it [29].

Most previous studies look at beliefs about the disease and protection and transmission measures by analyzing their relationship with protective behaviors [11,14,30,31] and, more rarely, this relationship is assessed regarding the psychological effects of an epidemic.

Because of all the above, when approaching the current pandemic situation by COVID-19, the information, knowledge, beliefs and concerns of the population should be taken into account given their influence on both the psychological and emotional impact this situation has on the population and on preventive behaviors. The objective of this study was to analyze psychological distress on a sample of the Spanish population during the beginning of the contagion curve in the COVID-19 pandemic, identifying the predictive nature that the information received, the preventive measures taken, the level of concern for transmitting the infection or being infected, the beliefs and the level of knowledge about the infection may have on psychological distress.

## 2. Materials and Methods 

### 2.1. Design Type

Cross-sectional observational study.

### 2.2. Sample

This study initially included a total of 4615 participants. In order to participate, it was necessary to comply with the following conditions: (i) living in Spain during the pandemic; (ii) being 18 years of age or older; and (iii) accepting the informed consent. A strict selection criterion was adopted, eliminating all questionnaires with an answer percentage of less than 99% (435 questionnaires), leaving 4180 questionnaires in the final sample. Questionnaires were received from the 50 Spanish provinces and the 2 small autonomous cities located in North Africa.

### 2.3. Questionnaires 

A specific questionnaire was developed for data collection, which included socio-demographic data, information received, prevention measures, beliefs, concerns, and population’s knowledge about COVID-19. Questions from similar previous studies [18] were adapted and new ones were added to meet the objectives of the study and cover the characteristics of the population.

As sociodemographic data, the variables collected were age, sex, level of studies, marital status, people with which they cohabited, and employment situation.

The information received was assessed by evaluating the number of sources of information and the hours spent listening, reading, or watching news about the pandemic per day. Items evaluating the accessibility, quantity, quality, and usefulness of information received through the media and official channels were included, with five categorized response options from very bad to very good. Questions about the amount of information received on symptoms, prognosis, treatments, routes of transmission, and preventive measures were added. A dichotomous response question (yes/no) was included to assess whether the person contrasted the information received with official sources.

Prevention measures were evaluated through questions with five answer options categorized from never to always regarding how often the following behaviors were performed: covering your mouth using your elbow when coughing or sneezing; avoiding sharing utensils (e.g., fork) during meals; washing hands with soap and water; washing hands with hydro-alcoholic solution; washing hands immediately after coughing, touching your nose or sneezing; washing hands after touching potentially contaminated objects; wearing a mask regardless of the presence or absence of symptoms; leaving at least a meter and a half of separation from others.

Beliefs and concerns about COVID-19 were assessed through 14 Likert-type answer questions from 1 to 10, a higher score meaning higher agreement. To assess participants’ knowledge, five basic questions on knowledge about COVID-19 regarding its transmission, symptoms, and prevention measures were included with “yes”, “no”, and “I don’t know” as answer options.

The questionnaire was pre-piloted by a panel of experts formed by psychologists, occupational doctors and nurses, epidemiologists, and public health experts. Subsequently, a piloting was carried out in which 57 people from different professions, educational levels, sex, age, and geographical areas of Spain participated. No comprehension issues or relevant incidents were identified.

Psychological adjustment was measured by the general health questionnaire (GHQ-12) [32], a tool used to assess mental health and psychological well-being. This consists of 12 items with four answer options; the first two are assigned a score of 0 points, and the last two are assigned a score of 1 point, so the total score ranges from 0 to 12. The questionnaire has been adapted and validated for the Spanish population, obtaining good internal consistency (Cronbach’s alpha coefficient of 0.86) and good psychometric properties [33]. The cut-off point set for the general population was three, considering psychological distress those with scores greater than or equal to 3 [34]. Cronbach’s alpha amounted to 0.851.

### 2.4. Procedure

Data were collected through an online questionnaire, the Qualtrics^®^ survey and storage platform. In this way, the confinement measures established during the pandemic did not interfere with the data collection process. For the sampling, the snowball method was chosen, involving professional colleges and associations, universities, and scientific societies in the process of disseminating the information, as well as through social networks and press. The questionnaires were collected between 26 March and 26 April. The health alert was decreed in Spain thirteen days before the start of the study.

### 2.5. Data Analysis

The analyses were performed using the SPSS statistical software (26.0) (IBM, Armonk, NY, USA). The presence or absence of psychological distress was assessed for each independent variable (information received, preventive measures taken, level of concern about transmitting the infection or getting infected, beliefs, and level of knowledge about the infection). Subsequently, bivariate analyses were performed, including Chi-squared test and student’s T-test for the independent variables, depending on their type. Crammer’s V and Cohen’s d effect size indexes were also calculated with the following cut-off points: 0 to 0.19, negligible; 0.20 to 0.49, small; 0.50 to 0.79, medium; from 0.80 on, high.

Then, with the aim of studying the predictive ability for psychological distress of the different sets of variables, logistic regression analyses (controlled by sex and age) were carried out including variables with *p* value <0.05. Finally, variables that manifested to have a predictive nature in each of the models were included in a global model (Model 5).

Odds ratios (ORs) were calculated with a 95% confidence interval.

### 2.6. Ethical Principles

The ethical principles set out in the Helsinki Declaration have been followed. The permission of the participants was obtained through an informed consent in which they expressed their voluntary desire to participate in the study. Data were recorded anonymously and treated confidentially. The study was authorized by the Research Ethics Committee of Huelva, belonging to the Andalusian Ministry of Health (PI 036/20). This study is integrated into a larger investigation that includes other variables on the psychological impact of the COVID-19 pandemic on the general population and on healthcare professionals. Some of the results that differ from the present study have already been published [35].

## 3. Results

### 3.1. Sociodemographic Data

The description of sociodemographic data is shown in Table 1. The sample consisted of a greater number of women (74.00%), most with university or higher education level (76.90%), married (57.80%), and a mean age of 40.26. Most of them were working away from home (44.70%), 20.70% at home via teleworking, and 34.50% were not working.

### 3.2. Information about COVID-19 and Psychological Distress

Data on information received on COVID-19 and its sources were analyzed. Participants were identified as consulting a mean of 3.31 (SD = 2.00) different sources of information, being social networks the most widely used (77.50%), followed by television (58.40%), official bodies or scientific societies websites (48.80%), friends or family (40.0%), online or printed press (34.90%), Google or other search engines (27.90%), radio (22.90%), and official phone numbers or information apps (13.30%). The results showed no statistically significant differences between this variable and the presence of psychological distress (t = 0.750, *p* = 0.453, Cohen’s d = 0.25).

Regarding the number of hours spent seeking information on COVID-19, the results were higher in the group that presented psychological distress, as compared to the group that did not present it (M = 4.53, SD = 3.29, and M = 3.80, SD = 2.62, respectively). Statistically significant differences were found between both groups (t = 7.498, *p* ≤ 0.001, Cohen’s d = 0.234, small effect size).

Taking into account the assessment made by the participants on the information provided by the media, participants with psychological distress rated the information provided by the media as more accessible (M = 4.02, SD = 0.94; t = −2.007, *p* = 0.045, Cohen’s d = 0.074), but of lower quality (M = 2.60, SD = 0.88; t = 3.290, *p* = 0.001, Cohen’s d = 0.113) and usefulness (M = 2.77, SD = 0.86; t = 2.261, *p* = 0.024, Cohen’s d = 0.078), as compared to the group that did not present this psychic morbidity. Statistically significant differences were also found in the assessment of the information provided by official means in terms of quantity (t = 2.004, *p* = 0.045, Cohen’s d = 0.071) and usefulness (t = 2.261, *p* = 0.024, Cohen’s d = 0.078), with a negligible effect size. In both cases, the scores were lower in the group of subjects who presented psychological distress (M = 3.39, SD = 0.98 and M = 2.98, SD = 1.01, respectively), as compared to the group which did not present psychological distress (M = 3.46, SD = 0.98, and M = 3.06, SD = 1.05).

Finally, and taking into account the assessment of the amount of information available on COVID-19, the results showed statistically significant differences in symptoms (t = 3.025, *p* = 0.003, Cohen’s d = 0.097), preventive measures (t = 2.749, *p* = 0.006, Cohen’s d = 0.093), transmission routes (t = 2.487, *p* = 0.013, Cohen’s d = 0.085), prognosis (t = 5.415, *p* = 0.001, Cohen’s d = 0.189), and treatment (t = 4.379, *p* ≤ 0.001, Cohen’s d = 0.149), with negligible effect sizes. For all cases, the group of subjects with psychological distress had a lower mean score (Table 2).

### 3.3. Preventive Measures and Psychological Distress

When analyzing the frequency of use of the recommended preventive measures (Table 3), the most common ones reported by participants have been washing hands with soap and water (M = 4.73, SD = 0.52), washing hands after touching potentially contaminated objects (M = 4.58, SD = 0.71), leaving at least a meter and a half of separation from others (M = 4.35, SD = 0.75), and avoiding sharing utensils during meals (M = 4.32, SD = 1.13). The last most commonly adopted measure was “wearing a mask regardless of the presence or absence of symptoms” (M = 3.12, SD = 1.53).

Statistically significant differences were found in terms of the use of preventive measures and the development of psychological distress. Seven of the eight measures showed significant differences (*p* = 0.05 in all cases), with effect sizes ranging from negligible to small. In each of them, the mean score obtained was higher in the group of subjects who presented psychological distress (Table 3). The only exception was in the preventive measure “leaving at least a meter and a half of separation from others”, where this group of participants obtained a lower mean score (M = 4.33, SD = 0.75), as compared to the group which did not present psychological distress (M = 4.40, SD = 0.76).

### 3.4. Concerns about COVID-19 and Psychological Distress

In response to concerns about COVID-19 (Table 4), participants expressed that being a transmitter of the infection was their main concern (M = 9.20, SD = 1.52), followed by the degree of general concern about COVID-19 (M = 8.20, SD = 1.73), and the degree of concern about becoming infected was in the last place (M = 7.37, SD = 2.41). The results showed statistically significant differences between both groups of subjects for all the variables (*p* < 0.001 in all cases), with small effect sizes. In this regard, the group of patients with psychological distress had higher scores (M = 9.33, SD = 1.88; M = 8.43, SD = 1.59, and M = 7.63, SD = 2.29, respectively), as compared to the group that did not present this psychic morbidity (M = 8.87, SD = 1.78; M = 7.62, SD = 1.94, and M = 7.37, SD = 2.41, respectively).

### 3.5. Beliefs and Knowledge about COVID-19 and Psychological Distress

Information on the relationship between beliefs and knowledge about COVID-19 and the presence of psychological distress is presented in Table 5.

In view of the participants’ beliefs on COVID-19, those who presented a higher score have been related with the need to provide a psychological support service to both the persons and families affected by the virus (M = 9.40, SD = 1.26), and with the professionals and volunteers who are directly involved in the health crisis (M = 9.2, SD = 1.48). Lastly, there are also beliefs about the infection having serious consequences for the participant’s health (M = 6.09, SD = 2.39), about being at risk of being infected (M = 6.49, SD = 2.71), about the level of confidence in the ability for diagnosing COVID-19 disease by the healthcare system (M = 6.94, SD = 2.27), and related to the difficulty of treatment of the infection (M = 6.97, SD = 2.02).

When assessing the relationship between beliefs on COVID-19 and the presence of psychological distress, the results showed statistically significant differences for all the variables (*p* < 0.001 in all cases), with effect sizes ranging from negligible to small. The group of subjects with psychological distress stated to have lower chances of survival if infected by the virus (M = 7.99, SD = 1.89), less confidence in the ability for diagnosing the disease of both health professionals (M = 8.27, SD = 1.78) and the healthcare system (M = 6.82, SD = 2.27), as well as perceiving less effectiveness of the preventive measures carried out (M = 7.88, SD = 1.77), as compared to the group of subjects who did not present psychological distress (M = 8.31, SD = 1.84; M = 8.52, SD = 1.79; M = 7.25, SD = 2.23; M = 8.10, SD = 1.75, respectively). However, beliefs regarding the risk of becoming infected (M = 6.81, SD = 2.65), that the infection would have serious consequences for the participant’s health (M = 6.26, SD = 2.35), and the difficulty of treatment of the infection (M = 7.08, SD = 1.98) were higher in the group of subjects with psychological distress, as compared to the group that did not present this psychic morbidity (M = 5.66, SD = 2.70; M = 5.67, SD = 2.45; M = 6.70, SD = 2.10, respectively). Similarly, when subjects were asked whether they felt it necessary to offer psychological support to professionals and volunteers who are directly involved in the health crisis, to individuals and families affected by COVID-19, as well as to the general population, the group with psychological distress showed significantly higher scores (Table 5).

Finally, most participants showed a high level of knowledge about COVID-19. Thus, most correctly answered questions were related with the need to isolate infected people (99.30%), transmission routes (97.00%), the incubation period (91.80%), and on the infective capacity of asymptomatic people (83.20%). However, only 6.60% correctly answered questions related to the symptoms of the virus. No statistically significant association was found between any variables on the level of knowledge about COVID-19 and the presence of psychological distress (*p* > 0.05 in all cases).

### 3.6. Prediction of Psychological Distress

Logistic regression models are displayed in Table 6.

With Model 1, which included variables related to COVID-19 information, 10.03% of explained variance was obtained (χ² = 310.604, *p* < 0.001). Those participants who spent a greater number of hours consulting information related to COVID-19 (OR = 1.088, 95% CI = [1.059, 1.119]), who expressed a greater assessment of the accessibility of the information provided by the media (OR = 1.161, 95% CI = [1.068, 1.263]), and a lower assessment of the amount of information on the prognosis of the disease (OR = 0.883, 95% CI = [0.800, 0.974]) were more likely to suffer psychological distress. This model provided a sensitivity of 95.60% and a specificity of 15.60%, with a total of 73.3% of the sample correctly classified.

The variables related to preventive measures are detailed in Model 2. The predictive ability of this model was 10.10% (χ² = 304.301, *p* < 0.001), classifying 73.10% of subjects correctly (94.10% sensitivity and 20.80% specificity). Participants who performed the preventive measures of washing hands with hydroalcoholic solution more frequently (OR = 1.140, 95% CI = [1.070, 1.215]) and wearing a mask regardless of the presence or absence of symptoms (OR = 1.136, 95% CI = [1.080, 1.196]) were more likely to develop psychological distress. However, those subjects who most frequently leave at least a meter and a half of separation from others had a lower probability of developing this psychic morbidity (OR = 0.881, 95% CI = [0.799, 0.972]).

Model 3, which is related with concerns about COVID-19, showed a predictive ability of 15%, higher than the previous models (χ² = 458.100, *p* < 0.001), correctly classifying 73.60% of participants (94.10% sensitivity and 20.80% specificity). Those subjects with a higher degree of concern about COVID-19 were 1.098 times more likely to suffer psychological distress (95% CI = 1.058, 1.139). Similarly, participants with a higher degree of concern about becoming infected with the virus were 1.233 times more likely to develop psychological distress (95% CI = 1.173, 1.296).

With Model 4, related with the participants’ beliefs about COVID-19, an explained variance percentage of 17.60% was obtained (χ² = 542.134, *p* < 0.001), the highest of the presented models. This model provided sensitivity and specificity values of 93.80% and 26.20%, respectively, with a percentage of correctly classified subjects of 74.90%. Subjects who believed they could survive COVID-19 in the event of infection (OR = 0.925, 95% CI = [0.882, 0.969]), with a higher level of confidence in the ability for diagnosing the disease by the health system (OR = 0.927, 95% CI = [0.891, 0.965]), and with greater confidence in the effectiveness of preventive measures (OR = 0.948, 95% CI = [0.906, 0.992] were less likely to develop psychological distress. Likewise, participants who considered to have an increased risk of being infected (OR = 1.149, 95% CI = [1.118, 1.182]), to be likely to suffer greater consequences for their health in the event of becoming infected (OR = 1.061, 95% CI = [1.022, 1.102]), and further expressed the need to provide psychological support to the general population (OR = 1.135, 95% CI = [1.089, 1.183]) were more likely to develop this psychic morbidity.

Finally, Model 5 (global model), where variables that showed a predictive ability in previous models were included, presented a predictive ability of 21.90%, correctly classifying 75.70% of participants (93.40% sensitivity and 30.00% specificity). The variables that showed a predictive ability were sex, age, number of hours consulting information on COVID-19, assessment of the information provided by the media in terms of accessibility, assessment of the information available on the prognosis of the disease, washing hands with hydroalcoholic solution, degree of concern about COVID-19, degree of concern to become infected, belief about the likelihood of survival if infected, level of confidence in the diagnostic ability of the health system, risk of getting infected, the belief about the effectiveness of preventive measures, and the need to offer psychological support to the general population (Table 5).

The variables that showed a higher weight, with ORs greater than 1, were being female (OR = 1.854, 95% CI = [1.575, 2.184]), degree of concern about COVID-19 (OR = 1.223, CI 95% = [1.161, 1.287]), assessment of the information provided by the media in terms of accessibility (OR = 1.163 95% CI = [1.072, 1.236]), the belief about the risk of being infected (OR = 1.112, 95% CI = [1.079, 1.147]), the need to offer psychological support to the general population (OR = 1.142, 95% CI = [1.102, 1.183]), washing hands with hydroalcoholic solution (OR = 1.071, 95% CI = [1.002, 1.144]), the degree of concern about becoming infected (OR = 1.054, 95% CI = [1.012, 1.097]), and the number of hours spent consulting information on COVID-19 (OR = 1.047, 95% CI = [1.018, 1.077]).

## 4. Discussion

The results indicate that the population is actively looking for information on COVID-19. Participants consulted several sources of information, with social media being the most common one. People with psychological distress spent more hours a day looking for information, and considered it more accessible, albeit of worse quality and usefulness. In addition, the information provided by the official channels in terms of quantity and usefulness was valued with lower scores.

Choosing the internet as the main source of information is consistent with the results of previous studies [36]. The lack or inadequacy of the information has been identified as a stressor during this pandemic, which leads the population to find answers to their concerns [37]. The Internet is currently the leading source of information worldwide, and users approach it as the first means of communication and information for health-related issues [38]. Abd-Alrazaq et al. analyzed the contents of the social network Twitter that were related to COVID-19 and identified the topics that most affected users: the origin of the virus; routes of transmission; impact on people and countries (death toll, stress and fear, travels, economy, and xenophobia), and risk and spread control measures [39]. Another similar analysis of the content on social networks related to COVID-19 grouped the topics of interest into five categories: (1) update of new cases and their impact; (2) first-line reports on the epidemic and its prevention measures; (3) expert opinions on the outbreaks of the infection; (4) frontline health services; and (5) global reach of the epidemic and identification of suspected cases [40].

The concern and need for information of the population is reflected in the use of social networks. The study conducted by Li et al. revealed that, following the outbreak of COVID-19, the expression of negative feelings on social media such as anxiety, depression or outrage increased significantly. Users expressed greater concern for their health and that of their families, and less interest in leisure and friends [41]. On the other hand, Zhao et al. identified an evolution of the content on social networks from the beginning of the health crisis, being it from negative to neutral, and a progressive increase in the expression of positive emotions [40].

The use of the internet as a source of health-related information also implies a risk. As Cuan-Baltazar et al. state, the quality of the information available on the internet on COVID-19 does not meet the quality criteria and may lead to a worrying situation of misinformation to the non-healthcare related population who do not have criteria to discriminate [38]. A recent critical analysis of the contents of the websites that disclosed the preventive measures before COVID-19 revealed that, in most cases, the information was ambiguous and not in line with WHO recommendations. Less than half of participants reported on the proper use of masks and that the most correct information was provided by official bodies’ websites [42].

Regarding adherence to preventive measures, the behavior that participants stated most often was hand washing. Participants with psychological distress performed preventive measures more frequently than those without distress, except for leaving a meter and a half of separation from others. The high adhesion obtained to hand washing and respiratory hygiene measures is consistent with results from previous studies [26,43,44,45]. These measures are in line with WHO recommendations [8] and are among the most suggested ones to deal with the COVID-19 pandemic [8,46]. The practice of preventive measures was associated with the perception of risk of coronavirus infection [47].

The results of this study coincide with Wang et al. by identifying the flattering influence of psychological distress on preventive behaviors regarding the spread of COVID-19 [18]. In relation to depressive symptomatology, studies show that the implementation of more precautionary behaviors and greater social distance is associated with a higher level of anxiety [31,48]. Still, authors like Cowling et al. found that a lower use of hygiene measures and greater social distancing have been associated with increased anxiety [30]. It seems clear that social distancing is related to the psychological impact, leading to greater symptomatology. What does not seem to be so clear is the role of individual protective measures, which may be mediated by other variables such as the perceived risk or vulnerability of getting infected.

The results of the present study indicate a high level of public concern regarding COVID-19, especially for those participants who presented psychological distress. These results are supported by findings from similar studies that reveal a high public concern about the COVID-19 pandemic [36,41,49,50,51], calling it terrifying [52]. The cause of most concern among participants was the possibility of being a transmitter. However, in similar studies, the main concerns were the infection of a family member [18] or getting infected with coronavirus [36,49]. According to Cori et al., 2020 an individual’s risk perception is modulated by four elements: voluntariness, knowledge, visibility, and trust; regarding the latter, the unknown risks are perceived as more threatening [53]. However, Wolf et al. identified that people with less health knowledge considered themselves less likely to get infected with coronavirus [49]. The uncertainty expressed by the population to this new disease manifests itself with situations of anxiety, depression, and sleep disorders [20]. People in confinement, as a measure of containment in the face of the spread of the COVID-19 epidemic, reported having low sleep quality aggravated by anxiety and stress [54].

Faced with the situation of concern and uncertainty generated by the health crisis, studies have described the level of public confidence regarding the measures put in place by their governments. Some authors identified that most participants felt that the country could win the battle against coronavirus [55], were satisfied with the epidemic control measures taken [52] and were motivated to follow the government’s recommendations on quarantine and social distancing [51]. On the contrary, the study of Wolf et al. revealed that half of the participants did not trust their government’s ability to contain the COVID-19 outbreak, and people with less health knowledge were more likely to rely on the government’s actions [49]. McFadden’s results point to health workers as the better able to lead the COVID-19 pandemic response strategy, according to the population’s assessment [56]. In order to face the concerns about COVID-19, coping strategies such as focusing on the problem and seeking alternatives, receiving emotional support and positive assessment of the situation [57], and doing physical exercise are recommended [58].

Participants in this study showed a high level of knowledge about COVID-19, except for their symptoms. These results support those obtained in previous similar studies that describe a good degree of knowledge on the part of the population, albeit disparately. On the one hand, there are authors who reported that participants were generally aware of coronavirus [55], its symptoms [49,59], routes of transmission [52,59], and preventive measures [49,51]. On the other hand, some authors identified knowledge gaps related to symptoms [51] and preventive measures [59]. Regarding university students, a good level of knowledge about the COVID-19 pandemic and its preventive measures has been described, especially among students attending life sciences degree courses [60]. Knowledge of the COVID-19 pandemic has been associated with willingness towards preventive measures and less confidence in the success of the fight against the virus [55]. Abdelhafiz et al. found that older people with low education, lower income, and living in rural areas tend to have less knowledge about the COVID-19 pandemic [36]. However, the profile of the person with little knowledge described by Zhong et al. is young women with low level of education, who are unemployed or students [55].

In this study, sex, accessibility to information, hours spent looking for information about coronavirus, degree of concern, belief of becoming infected, washing hands with hydroalcoholic solution, and perceived need for psychological help have been identified as factors with higher predictive weight of psychological distress. These results are in line with previous studies which have identified an association between female gender, negative affect, and detachment and higher levels of depression, anxiety, and stress [61]. Quarantine as a measure of containment has negative psychosocial consequences such as symptoms of depression, anxiety, anger, stress, post-traumatic stress, social isolation, loneliness, and stigmatization [62]. Psychological support interventions are needed to approach the situation, as the absence of psychological support is associated with higher levels of anxiety and depression [63].

Bäuerle et al. proposed a self-guided tool to promote psychological well-being based on mindfulness to reduce stress in the face of the COVID-19 crisis, to enhance coping strategies, perceive self-effectiveness, and mobilize personal resources [64]. Several community care initiatives have been described, which have been managed by mental health professionals who act as counsellors and by volunteer staff. Phone calls and Apps provide support, advice, and training to address the psycho-emotional impact of the pandemic [65,66].

The cross-sectional observational design of the study can be considered a limitation as it offers a photograph of what is happening at a precise time and does not allow inferring that such levels of psychological distress occur equally throughout the pandemic period. However, being able to obtain data at the time of the rise of the contagion curve is precisely what gives greater value to the study. The sample collection was not randomized and there were more women than men, factors that were compensated with a large sample and a representation of all the provinces and autonomous cities. It is difficult to compare the results between countries because confinement measures or cessation of labor activities differ greatly among them. Further study is planned to check for the effects at different stages of the pandemic.

## 5. Conclusions

This study revealed a strong psychological impact on the population as a result of the COVID-19 pandemic. The results describe a population profile that searches for information about the coronavirus by consulting various sources of information, although social media was the most widely used. With regard to adherence to preventive measures, the behavior that participants most often reported was hand washing and respiratory hygiene. The results of our study indicate that the population has a high level of concern and knowledge in relation to COVID-19, and this is especially true for those who presented psychological distress. Logistic regression analyses, on the other hand, have shown an adequate adjustment for the most part and an explained variance that exceeds 20% in the global model, being sex, degree of concern about the virus, getting infected, accessibility to information, number of hours looking for information, hand washing with hydroalcoholic solution, amount of information available on the prognosis of the disease, beliefs about the risk of infection, or need for psychological care for the population, among others, the predictors with greatest weight for psychological distress.

These results could help design more effective strategies for a psycho-emotional approach of the population in similar health crisis situations. Interventions aimed at the psychological well-being of the population are necessary to meet the needs of their reality.

## Figures and Tables

**Table 1:** Sociodemographic characteristics (*n* = 4180).

Variables	*N* (%)
Sex	
Male	1088 (26.00)
Female	3092 (74.00)
Age [mean (SD)]	40.26 (13.18)
Marital Status	
Single	1419 (33.90)
Married or living as a couple	2416 (57.80)
Separated/Divorced	296 (7.10)
Widowed	49 (1.20)
Educational level	
Primary education	57 (1.40)
Lower secondary education	65 (1.60)
Upper secondary education	838 (20.00)
University or higher	3212 (76.90)
Employment status	
Working away from home	1869 (44.70)
Working from home	867 (20.70)
Not working	1444 (34.50)

**Table 2:** Association between information about coronavirus disease (COVID-19) and psychological distress during the pandemic (*n* = 4180).

Variables	*M* (SD)	Psychological Distress		*t*	*p*	Effect Size
		No (*N* = 1171)	Yes (*N* = 3009)			
Number of consulted sources of information	3.31 (2.00)	3.28 (1.97)	3.33 (2.01)	−0.750	0.453	0.025
Number of hours looking for information related with COVID-19	4.32 (3.14)	3.80 (2.62)	4.53 (3.29)	−7.498	<0.001	0.234
Verification of the veracity of the information with official sources						
No	798 (19.10)	18.40	19.30	0.438	0.508	0.010
Yes	3382 (80.90)	81.60	80.70			
Assessment of the information about COVID-19 provided by the media in terms of *						
Accessibility	4.00 (0.95)	3.95 (0.96)	4.02 (0.94)	−2.007	0.045	0.074
Quantity	4.35 (0.90)	4.32 (0.90)	4.36 (0.90)	−1.060	0.289	0.044
Quality	2.62 (0.89)	2.70 (0.91)	2.60 (0.88)	3.290	0.001	0.113
Usefulness	2.80 (0.87)	2.86 (0.90)	2.77 (0.86)	2.804	0.005	0.103
Assessment of the information about COVID-19 provided by the official means in terms of *						
Accessibility	3.39 (0.99)	3.43 (0.99)	3.38 (0.98)	1.400	0.162	0.051
Quantity	3.41 (0.98)	3.46 (0.98)	3.39 (0.98)	2.004	0.045	0.071
Quality	2.89 (1.04)	2.91 (1.05)	2.88 (1.04)	0.951	0.342	0.029
Usefulness	3.00 (1.02)	3.06 (1.05)	2.98 (1.01)	2.261	0.024	0.078
Assessment of the quantity of information about COVID-19 in terms of *						
Symptoms	3.80 (1.13)	3.88 (1.14)	3.77 (1.13)	3.025	0.003	0.097
Preventive measures	3.80 (1.18)	3.88 (1.17)	3.77 (1.19)	2.749	0.006	0.093
Infection routes	3.72 (1.17)	3.79 (1.17)	3.69 (1.17)	2.487	0.013	0.085
Prognosis	3.20 (1.17)	3.36 (1.18)	3.14 (1.16)	5.415	<0.001	0.189
Treatment	2.75 (1.21)	2.88 (1.24)	2.70 (1.20)	4.379	<0.001	0.149

* Note: Likert-type scale from 0 (very low) to 5 (very high).

**Table 3:** Association between preventive measures and psychological distress during the COVID-19 pandemic (*n* = 4180).

Preventive Measures	*M* (SD)	Psychological Distress		*t*	*p*	Effect Size
		No (*N* = 1171)	Yes (*N* = 3009)			
Covering mouth with elbow when coughing or sneezing	4.25 (0.98)	4.19 (0.92)	4.27 (0.86)	−2.836	0.005	0.091
Avoiding sharing utensils (e.g., fork) during meals	4.32 (1.13)	4.26 (1.19)	4.34 (1.10)	−2.089	0.037	0.071
Washing hands with soap and water	4.73 (0.52)	4.69 (0.56)	4.74 (0.50)	−2.380	0.017	0.097
Washing hands with hydroalcoholic solution	3.49 (1.25)	3.26 (1.29)	3.58 (1.22)	−7.592	<0.001	0.258
Washing hands immediately after coughing, sneezing or rubbing nose	3.64 (1.14)	3.54 (1.17)	3.68 (1.12)	−3.486	0.001	0.123
Washing hands after touching potentially contaminated objects	4.58 (0.71)	4.57 (0.71)	4.58 (0.71)	−0.187	0.852	0.014
Wearing a mask regardless of the presence or absence of symptoms	3.12 (1.53)	2.85 (1.54)	3.23 (1.51)	−7.280	<0.001	0.250
Leaving at least a meter and a half of separation from others	4.35 (0.75)	4.40 (0.76)	4.33 (0.75)	2.602	0.009	0.093

Note: Likert-type scale from 0 (never) to 5 (always).

**Table 4:** Association between concerns about COVID-19 and psychological distress during the pandemic (*n* = 4180).

Concerns about COVID-19	*M* (SD)	Psychological Distress		*t*	*p*	Effect Size
		No (*N* = 1171)	Yes (*N* = 3009)			
Degree of concern about COVID-19	8.20 (1.73)	7.62 (1.94)	8.43 (1.59)	−12.596	<0.001	0.478
Degree of concern about being a transmitter	9.20 (1.52)	8.87 (1.78)	9.33 (1.38)	−7.989	<0.001	0.306
Degree of concern about getting infected	7.37 (2.41)	6.69 (2.55)	7.63 (2.29)	−10.987	<0.001	0.397

Note: Likert-type scale from 1 (not concerned at all) to 10 (very concerned).

**Table 5:** Association between beliefs and knowledge about COVID-19 and psychological distress during the pandemic (*n* = 4180).

Variables	*M* (SD)	Psychological Distress		*t*	*p*	Effect Size
		No (*N* = 1171)	Yes (*N* = 3009)			
Beliefs about COVID-19 *						
Likelihood of survival if infected	8.08 (1.88)	8.31 (1.84)	7.99 (1.89)	4.985	<0.001	0.171
Degree of confidence in the diagnostic ability:						
Of health professionals	8.34 (1.78)	8.52 (1.79)	8.27 (1.78)	4.104	<0.001	0.140
Of the health system	6.94 (2.27)	7.25 (2.23)	6.82 (2.27)	5.599	<0.001	0.252
Risk of getting infected	6.49 (2.71)	5.66 (2.70)	6.81 (2.65)	−12.559	<0.001	0.432
The infection would have serious consequences for the health	6.09 (2.39)	5.67 (2.45)	6.26 (2.35)	−7.057	<0.001	0.248
The infection is hard to treat	6.97 (2.02)	6.70 (2.10)	7.08 (1.98)	−5.316	<0.001	0.189
Effectiveness of preventive measures	7.94 (1.77)	8.10 (1.75)	7.88 (1.77)	3.646	<0.001	0.125
Need to offer psychological support to:					<0.001	
Professionals and voluntary staff	9.28 (1.48)	8.96 (1.81)	9.40 (1.30)	−7.532	<0.001	0.301
Persons and families affected by COVID-19	9.40 (1.26)	9.11 (1.57)	9.52 (1.10)	−8.049	<0.001	0.328
The general population	8.53 (2.03)	7.89 (2.40)	8.78 (1.85)	−11.590	<0.001	0.441
**Knowledge about COVID-19**						
About the incubation period						
Right	3836 (91.80)	91.20	92.0	0.691	0.406	0.013
Wrong or do not know	344 (8.20)	8.80	8.00			
About the most common symptoms						
Right	274 (6.60)	6.90	6.40	0.348	0.555	0.009
Wrong or do not know	3906 (93.40)	93.10	93.60			
About isolation of infected people						
Right	4150 (99.30)	99.30	99.30	0.027	0.869	0.003
Wrong or do not know	30 (0.70)	0.70	0.70			
About transmission routes						
Right	4053 (97.00)	97.30	96.80	0.516	0.473	0.011
Wrong or do not know	127 (3.00)	2.70	3.20			
About the infective capacity of asymptomatic people						
Right	3479 (83.20)	81.50	83.90	3.614	0.057	0.029
Wrong or do not know	701 (16.80)	18.50	16.10			

Note: * Likert-type scale from 1 to 10.

**Table 6:** Logistic regression models on psychological distress by set variables.

-	Variables	Model 1 OR (95% CI) Information	Model 2 OR (95% CI) Preventive Measures	Model 3 OR (95% CI) Concerns	Model 4 OR (95% CI) Beliefs and Knowledge	Model 5 Global Model OR (95% CI)
		R² = 0.103 (95.6/15.6%)	R² = 0.101 (96.2/13.6%)	R² = 0.150 (94.1/20.8%)	R² = 0.176 (93.8/26.2%)	R² = 0.219 (93.4/30.0%)
**Socio-demographic**	Sex (ref. males)	2.416 ** (2.075, 2.812)	2.236 ** (1.917, 2.608)	2.179 ** (1.866, 2.543)	1.983 ** (1.690, 2.327)	1.854 ** (1.575, 2.184)
	Age	0.980 ** (0.975, 0.986)	0.977 ** (0.971, 0.982)	0.970 ** (0.964, 0.975)	0.974 ** (0.969, 0.980)	0.971 ** (0.965, 0.977)
**Information**	Number of hours consulting information	1.088 ** (1.059, 1.119)	NA	NA	NA	1.047 * (1.018, 1.077)
	Assessment of the information provided by the media in terms of					
	Accessibility	1.161 ** (1.068, 1.263)	NA	NA	NA	1.163 ** (1.072, 1.236)
	Quality	0.919 (0.825, 1.024)	NA	NA	NA	NA
	Usefulness	1.021 (0.913, 1.142)	NA	NA	NA	NA
	Assessment of the information provided by official means in terms of					
	Quantity	0.977 (0.900, 1.061)	NA	NA	NA	NA
	Usefulness	0.921 (0.845, 1.004)	NA	NA	NA	NA
	Assessment of the quantity of information available in terms of					
	Symptoms	0.993 (0.901, 1.094)	NA	NA	NA	NA
	Preventive measures	0.951 (0.864. 1.046)	NA	NA	NA	NA
	Infection routes	1.039 (0.943, 1.145)	NA	NA	NA	NA
	Prognosis	0.883 * (0.800, 0.974)	NA	NA	NA	0.897 ** (0.835, 0.963)
	Treatment	0.987 (0.911, 1.070)	NA	NA	NA	NA
**Preventive measures**	Covering mouth with elbow when coughing or sneezing	NA	0.933 (0.854, 1.019)	NA	NA	NA
	Avoiding sharing utensils (e.g., fork) during meals	NA	1.021 (0.958, 1.088)	NA	NA	NA
	Washing hands with soap and water	NA	1.019 (0.882, 1.178)	NA	NA	NA
	Washing hands with hydroalcoholic solution	NA	1.140 ** (1.070, 1.215)	NA	NA	1.071 * (1.002, 1.144)
	Washing hands immediately after coughing, sneezing or rubbing your nose	NA	1.048 (0.973, 1.129)	NA	NA	NA
	Wearing a mask regardless of the presence or absence of symptoms	NA	1.136 ** (1.080, 1.196)	NA	NA	1.038 (0.983, 1.097)
	Leaving at least a meter and a half of separation from others	NA	0.881 * (0.799, 0.972)	NA	NA	0.908 (0.819, 1.008)
**Concerns**	Degree of concern about COVID-19:	NA	NA	1.098 ** (1.058, 1.139)	NA	1.223 ** (1.161, 1.287)
	Degree of concern about being a transmitter	0.993 (0.901, 1.094)	NA	1.041 (0.992, 1.093)	NA	NA
	Degree of concern about getting infected	0.951 (0.864. 1.046)	NA	1.233 ** (1.173, 1.296)	NA	1.054 * (1.012, 1.097)
**Beliefs and knowledge**	Likelihood of surviving if infected	NA	NA	NA	0.925 ** (0.882, 0.969)	0.933 * (0.889, 0.979)
	Degree of confidence in the diagnostic ability of:					
	Health professionals	NA	NA	NA	0.997 (0.945, 1.051)	NA
	The health system	NA	NA	NA	0.927 ** (0.891, 0.965)	0.933 ** (0.900, 0.966)
	Risk of getting infected	NA	NA	NA	1.149 ** (1.118, 1.182)	1.112 ** (1.079, 1.147)
	The infection would have serious health consequences	NA	NA	NA	1.061 ** (1.022, 1.102)	1.003 (0.963, 1.044)
	The infection is hard to treat	NA	NA	NA	1.026 (0.986, 1.068)	NA
	Effectiveness of preventive measures	NA	NA	NA	0.948 * (0.906, 0.992)	0.943 * (0.898, 0.990)
	Need for psychological support for:					
	Professionals and voluntary staff	NA	NA	NA	1.056 (0.990. 1.127)	NA
	Persons and families affected by COVID-19	NA	NA	NA	1.035 (0.955, 1.121)	NA
	The general population	NA	NA	NA	1.135 ** (1.089, 1.183)	1.142 ** (1.102, 1.183)

* *p* < 0.05; ** *p* < 0.01; NA: not applicable; R² = model explained variance (sensitivity/specificity); OR (95% CI): odds Ratio (confidence interval at the 95% level).
